# A New Genus *Bifidunguiglenea* gen. nov. Is Erected for the Species *Glenea gestroi* Gahan (Cerambycidae: Lamiinae: Saperdini)

**DOI:** 10.1371/journal.pone.0040768

**Published:** 2012-07-17

**Authors:** Mei-Ying Lin, Gérard Luc Tavakilian

**Affiliations:** 1 Institute of Zoology, Chinese Academy of Sciences, Chaoyang, Beijing, China; 2 Antenne IRD, Entomologie, Département de Systématique et Évolution, Muséum National d’Histoire Naturelle, Paris, France; Swedish University of Agricultural Sciences, Sweden

## Abstract

*Bifidunguiglenea* **gen. nov.** is erected for the species *Glenea gestroi* Gahan, 1894. *Bifidunguiglenea gestroi* (Gahan, 1894) **comb. nov.** is redescribed. The genitalia descriptions are reported for the first time and it is newly recorded from Thailand.

## Introduction

*Glenea* Newman, 1842 is one of the largest genera of Cerambycidae, including 36 subgenera and more than 850 species (according to Mr. Tavakilian’s database ‘Titan 2000’ and the senior author’s study) [1]. Most of the subgenera were erected for a small number of species, while the *Glenea* subgenus *Glenea* defined by Breuning (1956) [2] is extremely complex, including several very different groups which should be separated [1], [3], [4]. Gahan (1897) conducted an analysis “on the structure of the tarsal claws in the genus *Glenea*” [5]. He concluded that “in the female all the claws are simple” while in the male there were several different states. He also founded a new genus *Heteroglenea* for the reception of two species, *G. nigromaculata* Thomson and *G. glechoma* Pascoe for their exceptional tarsal claws [5]. In his revision of *Glenea* Newman, Breuning (1956–1958) did not follow Gahan’s concept but considered *Heteroglenea* as “überflüssiger Name”, and listed it in the synonyms of *Glenea* (*sensu stricto*), which was not clearly defined [4]. Based on external structural and genitalia characters, *Heteroglenea* Gahan was reinstated as a valid genus and redefined, including nine valid species [4].

*Glenea gestroi* Gahan, 1894 was described based on a single female from Myanmar, without a description of the tarsal claws [6]. Gahan (1897) did not include this species during his analysis on the structure of the tarsal claws in the genus *Glenea* [5], though its claws have an even more modified structure than his *Heteroglenea*. Pic (1928, 1930) also did not pay attention to the claws’ structure [6], [7]. Breuning (1956) synonymized *Glenea luteomaculata* Pic, 1928 [6] and *Glenea bicoloricornis* Pic, 1930 [7] with *Glenea gestroi* Gahan, 1894, without examining their claws and included this species in his *Glenea* (*sensu stricto*) [8]. We examined all the type specimens of the three names and a series of common specimens, and concluded that this species must be separated from *Glenea* Newman. *Bifidunguiglenea* **gen. nov.** is herein erected. It is possible there are more species that should be transferred from *Glenea* or other genera, and there might be some new species unknown to us, so this paper is far from a complete revision.

## Results and Discussion

### Taxonomic Treatment

***Bifidunguiglenea*** **gen. nov.**

(urn:lsid:zoobank.org:act:ED00D869-55FD-461D-A4A2-C18D1C1CDF23).

Type species: *Bifidunguiglenea gestroi* (Gahan, 1894) **comb. nov.**

#### Etymology

This genus is separated from the huge genus *Glenea*, based on the bifid claws of both males and females. “Bifidungui” means bifid claws. Gender feminine.

#### Definition

**Figure 1:**
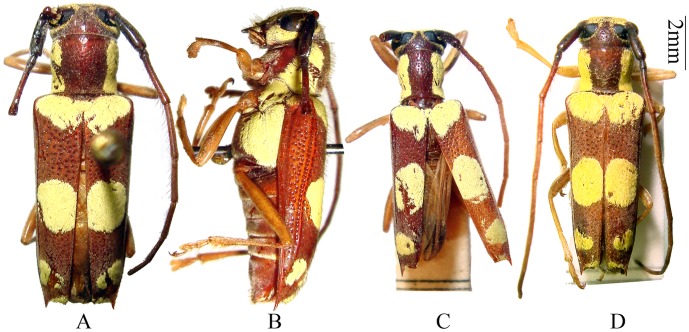
Habitus, *Bifidunguiglenea gestroi* (Gahan, 1894) comb. nov. (A–B) holoype of *Glenea gestroi* Gahan, female, from Myanmar. (A) dorsal view. (B) lateral view. (C) holotype of *Glenea luteomaculata* Pic, male, from Vietnam. (D) holotype of *Glenea bicoloricornis* Pic, female, from Vietnam. Scale 2 mm.

**Figure 2:**
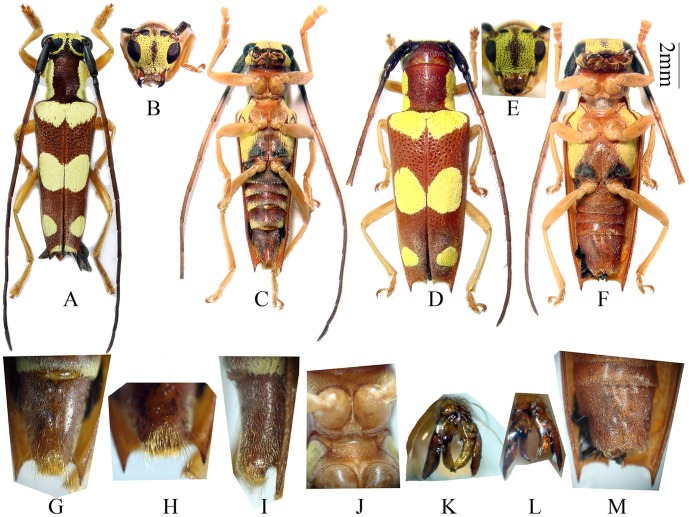
*Bifidunguiglenea gestroi* (Gahan, 1894) comb. nov. (A) male, from Thailand. (B–C) another male, from Thailand. (B) head, frontal view. (C) ventral view (D) female, from Thailand. (G–I & M) sternite VII (ventrite V). (G–I) male, showing the deep and semi-circular shaped notch. (G–H) ventral view. (I) lateral view. (M) female, ventral view, showing the middle shin groove. (J) showing the procoxal cavity closed posteriorly. (K–L) the structure of tarsal claw. (K) male, the inner teeth crossed. (L) female. Scale 2 mm. (G–M). not to scale.

Moderately sized (around 10 mm in length, Figures 1A–D, 2A–F). Head broad with tumid eyes, frons longer than broad (male) to broader than long (female), eyes deeply concave, inferior eyelobe subequal to (male) or much narrower (female) than half of frons (in front view). Antennae longer than body length, scape slightly expanded, without a ridge, the third or fourth antennomere longest (fourth subequal to third in female). Prothorax cylindrical, without lateral pronotal spine or tubercle. Elytra subparallel (slightly narrower apically), with two obtuse lateral carinae reaching the base but not the apex, truncated apically, elytral apex with a sharp spine at the outer angle. Procoxal cavity closed posteriorly (Figure 2J), metepisternum more than twice as wide anteriorly as posteriorly, middle tibiae grooved, hind femur reaching middle of fourth to middle of fifth abdominal segment. Both male and female have bifid claws on all the tarsi (Figures 2K–L).

**Figure 3:**
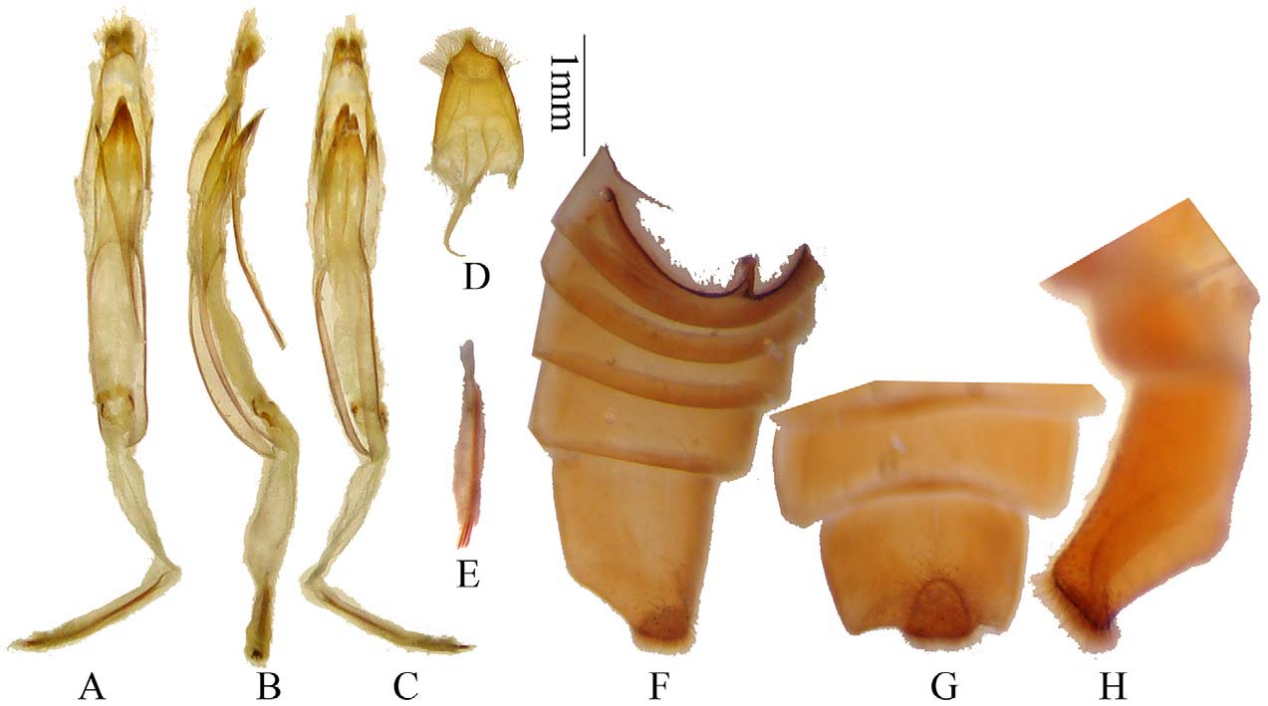
Terminalia of *Bifidunguiglenea gestroi* (Gahan, 1894) comb. nov., from Vietnam. (A–C) male genitalia. (A) ventral view, (B) lateral view, (C) dorsal view. (D) tergite VIII and sternites VIII & IX in ventral view. (E) rods of endophallus. (F–H) male, showing the deep and semi-circular shaped notch of sternite VII (ventrite V). Scale 1 mm. (F–H) not to scale.

**Figure 4:**
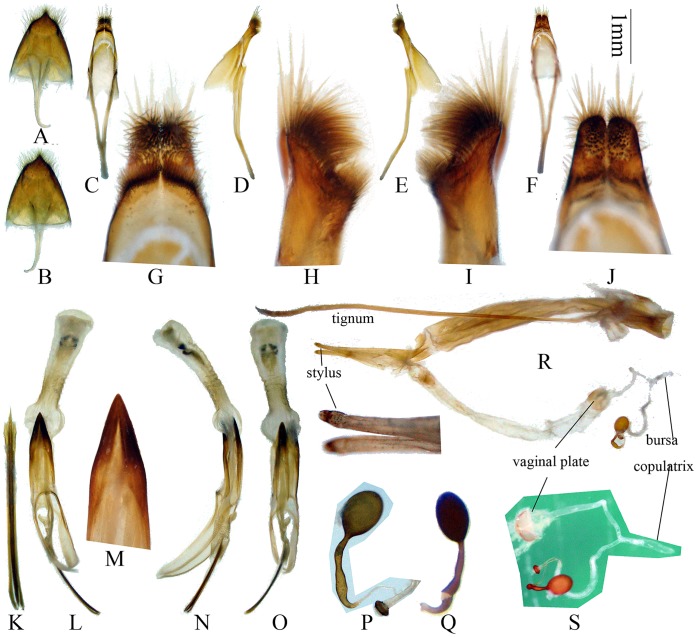
Terminalia of *Bifidunguiglenea gestroi* (Gahan, 1894) comb. nov., male from Thailand, female from Thailand and Vietnam. (C–O) male genitalia. (A–B) tergite VIII and sternites VIII & IX. (C–J) tegmen. (G–J) magnified, not to scale. (L–O) median lobe with median struts, with internal sac. (L) ventral view, (N) lateral view, (O) dorsal view. (M) showing apex of ventral plate and lateral lobes of tegmen. (K) rods of endophallus. (P–S) female genitalia. (P–Q). showing spermathecal capsule. (P) from Thailand. (Q) from Vietnam. Scale 1 mm. (G–J, K, M, P, Q, S) not to scale.

Male terminalia: Apex of tergite VIII with a protruding lobe in the middle (Figures 3D, 4A–B); lateral lobes of tegmen very short and stout, confluent with each other except apex (Figures 4G, 4J), ringed part elbowed in the widest portion, converging and elongated; median lobe plus median struts slightly curved (Figure 4N), a little longer than tegmen, internal sac with 3 rods. Female terminalia: spermathecal capsule can be divided into a basal stalk and an apical lobe (orb or ellipse), the basal stalk much longer than the apical lobe; tignum much longer than abdominal length in ventral view.

#### Diagnosis

Differs from *Glenea* Newman with claws of all the tarsi in both sexes bifid. Differs from *Heteroglenea* Gahan by all claws bifid (instead of only anterior claws bifid), elytron with two lateral carinae and elytral apex with sharp spine at the outer angle. The male genitalia of this genus are very different from all other saperdine members, with the lateral lobes of tegmen confluent to each other.

#### Remarks

Only one species is placed in this genus now, though we believe there are more species that should be removed from the genus *Glenea* or others and there are some undescribed species awaiting discovery. *Glenea pulchella* Pascoe, 1858 ( =  *G. vesta* Pascoe, 1866) and *G. vestalis* Heller, 1934 have been studied and we confirm they do not belong to this genus due to simple claws in both male and female and very different genitalia structures, though the body shape and maculae are very similar to *Bifidunguiglenea gestroi* (Gahan, 1894).

***Bifidunguiglenea gestroi*** **(Gahan, 1894) comb. nov.**

(Figures 1, 2, 3 and 4).

*Glenea gestroi* Gahan, 1894∶88, pl. 1, fig. 14 (Myanmar: Bhamò). [MCSNG] [6].

*Glenea luteomaculata* Pic, 1928∶30 (Vietnam: Tonkin). [MNHN] [7].

*Glenea bicoloricornis* Pic, 1930∶17 (Vietnam: Tonkin). [NMB] [8].

*Glenea (Glenea) gestroi*; Breuning, 1956∶197, pl. II, fig. 6 [9]; Breuning, 1966∶685 [10].

#### Redescription

Male (Figures 1C, 2A–C): length: 7.8–10.0 mm, humeral width: 2.2–2.7 mm. Female (Figures 1A–B, 1D, 2D–F): length: 9.5–11.1 mm, humeral width: 2.9–3.5 mm. Body reddish brown, thicky and strongly punctured. Antennae reddish brown, with first two antennomeres (always) and basal part of third antennomere (sometimes) darker. Head with lemon-yellow pubescence in front (except along the middle). Prothorax with a lemon-yellow band on each side; the upper borders of these bands are sub-parallel, so that the median dorsal space enclosed between them is nearly oblong in shape. Elytra with a basal transverse band, two large rounded spots at the middle which touch one another at the suture, two smaller spots before the apex, and two very small spots placed at the extreme apex, lemon-yellow. Mesepisternum, mesepimeron, metepisternum and lateral sides of metasternite similarly coloured (Figures 1B, 2C, 2F). Metasternite with two black spots just anterior hind coxal cavities (Figures 2C, 2F). Legs yellowish brown.

Head slightly broader than prothorax, feebly concave at vertex. Eyes deeply emarginate, inferior eye lobes slightly longer than (female), to 4 times as high as (male) genae below it, width subequal to (male) to much less than half of frons (female). Antennae longer than body; scape slightly thickened apically without cicatrix nor a ridge; antennomere ratio (male): 13∶3∶17∶18∶14∶13∶12∶11∶11∶10∶9; (female): 14∶3∶17∶17∶14∶12∶11∶10∶10∶9∶8. Prothorax almost as broad as long (male) or broader than long (female), swollen laterally before middle; disc convex and deeply and densely punctured. Elytra slightly narrowed apically; each with 2 humeral longitudinal ridges beginning at humeri and not reaching the apex; apices of the elytra with the inner angles slightly, the outer angles strongly and distinctly spined; surface with coarse and irregular punctures. Legs stout, hind femur reaching middle of fourth (female) to middle of fifth (male) abdominal segment, first hind tarsal segment longer than (male), or nearly as long as (female) following two segments combined. Apex of sternite VII (ventrite V) risen up with a deep and semi-circular shaped notch, filled with dense hairs (Figures 2G–H, 3F–H).

Male genitalia (Figures 3A–E, 4A–O): Tegmen length about 2.8 mm; lateral lobes (Figures 4G–J) very stout and confluent except apex, ventral face filled with dense setae, with two densely haired small lobes at the base (Figure 4G, in ventral view), apex rounded and with longer setae; ringed part elbowed in the widest portion, converging; basal piece not bifurcated distally (Figure 4F); median lobe plus median struts moderately curved (Figure 4N), a little longer than tegmen; the median struts about one half of the whole median lobe in length; dorsal plate not shorter than ventral plate (Figures 3B, 4N, 4O); apex of ventral plate narrowly pointed (Figure 4M); median foramen elongated; internal sac about 2 times as long as median lobe plus median struts in length, with 4 pieces of basal armature and 3 rods; rods each about 1.2 mm, shorter than half of tegmen. Ejaculatory duct single. Tergite VIII (Figures 3D, 4A–B) longer than broad, apex with a protruding lobe in the middle, setae fine and short.

Female genitalia (Figures 4P–S): Tignum much longer than abdomen in ventral view. We observed a 6.7 mm tignum for an adult with a 4.3 mm abdomen in ventral view. Stylus without setae. Spermathecal duct much longer than spermathecal capsule (Figure 4S); spermathecal gland has a strongly sclerotized broad ring (Figure 4P); spermathecal capsule can be divided into an apical orb and a long and curved basal stalk (Figures 4P–Q).

#### Diagnosis

This species somewhat resembles *Glenea vesta* Pascoe, 1866 but may be distinguished by its paler colour; the oblong form of the dorsal median brown space of the prothorax; the presence of two distinct spots, conjoined at the suture, which are placed at the middle of the elytra, and of a small transverse or slightly oblique spot at the extreme apex of each elytron. The abdomen is almost entirely reddish brown underneath [6].

Except for the difference of color and pubescent markings mentioned by Gahan (1894), the most important difference is the structure of tarsal claws, which separates them into different genera.

#### Remarks

*Glenea luteomaculata* Pic, 1928 and *Glenea bicoloricornis* Pic, 1930 were synonymized with *Glenea gestroi* Gahan, 1894 by Breuning (1956). We agree with Breuning after checking all the types. The Thailand material was determined by comparing their external characters and genitalia structures.

#### Distribution

(Map S1).

Myanmar, Thailand (**new country record**), Vietnam.

#### Type specimens examined

Holoype of *Glenea gestroi* Gahan, female, Birmania, Bhamò (in upper Burma), 1885.VIII, leg. Fea (MSCNG). Holotype of *Glenea bicoloricornis* Pic, female, Hoa Binh (NMB, ex Coll. Frey). Holotype of *Glenea luteomaculata* Pic, male, Tonkin, Hoa-Binh (MNHN, ex Coll. Clermont).

**Other specimens examined.** **Thailand:** 2 males 2 females, N Thailand, Chiang Rai prov., Wiang Pa Pao env., 2011.V.21–VI.10, leg. P. Viktora (CPV, with one male one female deposited in IZAS, ex CPV).

**Vietnam:** 2 males, Tonkin, Hoa Binh, one leg. Cooman (NMB, ex coll. Frey); 3 females, Tonkin occ. Reg. De Hoa Binh, 1918, leg. R. P. A. de Cooman (MNHN, ex Coll. R. Oberthür, 1952).

## Materials and Methods

Materials studied have been deposited in the following institutions, museums or personal collections: CPV - Collection of Petr Viktora, Kutná Hora, Czech Republic; IZAS - Institute of Zoology, Chinese Academy of Sciences, Beijing, China; MCSNG - Museo Civico di Storia Naturale «Giacomo Doria», Genova, Italy; MNHN - Muséum National d’Histoire Naturelle, Paris, France; NMB - Naturhistorisches Museum, Basel, Switzerland (Museum Frey, Tutzing).

The habitus photos were captured using a digital camera SONY DSC-T30. The genitalia photos were captured using same camera coupled to a Leica stereomicro-scope S8APO. Photos were edited using Adobe Photoshop CS2.

### Nomenclatural Acts

The electronic version of this document does not represent a published work according to the International Code of Zoological Nomenclature (ICZN), and hence the nomenclatural acts contained in the electronic version are not available under that Code from the electronic edition. Therefore, a separate edition of this document was produced by a method that assures numerous identical and durable copies, and those copies were simultaneously obtainable (from the publication date noted on the first page of this article) for the purpose of providing a public and permanent scientific record, in accordance with Article 8.1 of the Code. The separate print-only edition is available on request from PLoS by sending a request to PLoS ONE, Public Library of Science, 1160 Battery Street, Suite 100, San Francisco, CA 94111, USA along with a check for $10 (to cover printing and postage) payable to “Public Library of Science”.

In addition, this published work and the nomenclatural acts it contains have been registered in ZooBank, the proposed online registration system for the ICZN. The ZooBank LSIDs (Life Science Identifiers) can be resolved and the associated information viewed through any standard web browser by appending the LSID to the prefix “http://zoobank.org/”. The LSID for this publication is: (urn:lsid:zoobank.org:pub:9D2F587F-B6E8-4BCA-8394-797E62E4557A). The online version of this work is archived and available from the following digital repositories: PubMed Central, LOCKSS.

## Supporting Information

Map S1 **Known distribution points of** ***Bifidunguiglenea gestroi*** **(Gahan, 1894) comb. nov.**
(TIF)
