# Supplementary figures and images for: A New Genus Bifidunguiglenea gen. nov. Is Erected for the Species Glenea gestroi Gahan (Cerambycidae: Lamiinae: Saperdini)
Source: PLoS One. 2012 Jul 17;7(7):e40768. doi: 10.1371/journal.pone.0040768 (PMC3398961; doi:10.1371/journal.pone.0040768)

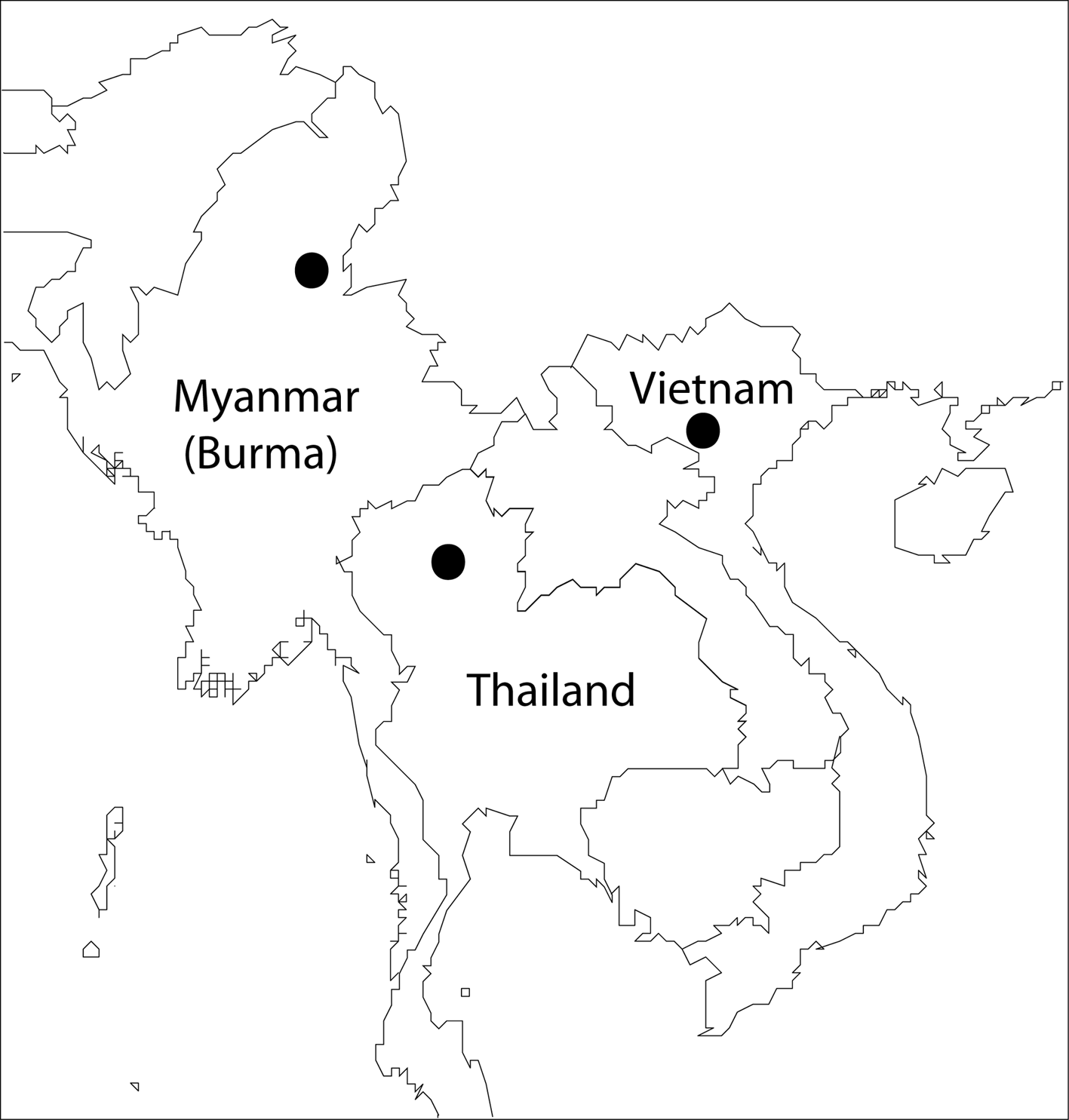

Supplement: Map S1 — Known distribution points of Bifidunguiglenea gestroi (Gahan, 1894) comb. nov. (TIF) [file pone.0040768.s001.tif]
